# A Review of *Franklinothrips vespiformis* (Thysanoptera: Aeolothripidae): Life History, Distribution, and Prospects as a Biological Control Agent

**DOI:** 10.3390/insects13020108

**Published:** 2022-01-18

**Authors:** Mubasher Hussain, Zhaohong Wang, Steven P. Arthurs, Jing Gao, Fengxian Ye, Lingling Chen, Runqian Mao

**Affiliations:** 1 Guangdong Key Laboratory of Animal Conservation and Resource Utilization, Guangdong Public Laboratory of Wild Animal Conservation and Utilization, Guangdong Engineering Research Center for Mineral Oil Pesticides, Institute of Zoology, Guangdong Academy of Sciences, Guangzhou 510260, China; wangzh@giz.gd.cn (Z.W.); gaoj@giabr.gd.cn (J.G.); yefengxian2021@126.com (F.Y.); c1627236708@126.com (L.C.); 2 BioBee USA, 5126 S. Royal Atlanta Dr, Tucker, GA 30084, USA; steven.arthurs@biobee.us

**Keywords:** ant-mimic, demography, habitat, *Franklinothrips*, prey specificity, predatory thrips

## Abstract

**Simple Summary:**

Predatory species are a small but significant part of the Thysanoptera, which is often overlooked. *Franklinothrips* are found throughout the tropics and are regarded as major natural enemies of thrips and other small arthropod prey. In this review, we summarized the geographical background, morphology, and prey associations, with an emphasis on *Franklinothrips vespiformis*, the most widely distributed predatory thrips species. This literature review could serve as a foundation for future research into *Franklinothrips* as biocontrol agents for economically important insect and mite pests in China.

**Abstract:**

Predatory species comprise a small but important and often overlooked component of the Thysanoptera. A case in point, the ant-mimicking *Franklinothrips* are widely distributed in the tropics and are considered important generalist natural enemies for thrips and some other small arthropod prey. *Franklinothrips* present an addition to biocontrol applications, i.e., greenhouse or commercial application for certain target pests and situations. Current knowledge, including distribution, biological features, life history pa rameters, prey specificity, host plant associations and lass production is yet insufficient to decide to what extent *Franklinothrips* could contribute for biological control programs. In this review, we summarized the geographical background, morphology, and prey associations, with a focus on *F. vespiformis*, the most widely distributed species of predatory thrips. This literature review serves as the basis for future research into the use of *Franklinothrips* as biocontrol agents for economically significant insect and mite pests in China and elsewhere.

## 1. Introduction

The Thysanoptera (thrips) constitute approximately 6500 species that are globally distributed and represent many of the smallest winged insects [1,2]. Several thrips species are globally important, due to their capacity to disperse through the plant trade and vector plant tospoviruses, which cause significant agricultural losses [3]. While most of the thrips are detritivores (mainly fungal feeders) and herbivores (feeders of flowers, fruits, and leaves) [4,5], approximately 300 species have evolved a predatory lifestyle [6].

Predatory thrips are known from several families. Surveys in three districts of northern Thailand revealed 10 species of predatory Phlaeothripidae in five genera, including *Aleurodothrips fasciapennis*, which were present throughout the year and contributed to pest control [7]. In USA, mite-predatory species *Scolothrips sexmaculatus* (Thripidae) and *Leptothrips mali* (Phlaeothripidae) are considered important biological control agents in almonds and apple orchards, respectively [8,9]. However, most of the predatory thrips species are confined within the Aeolothripidae. While most of the aeolothripids are generalist facultative predators of small arthropods and distributed in temperate regions [6], a few genera are native to the tropics and more specialized.

Species of *Franklinothrips* (Thysanoptera: Aeolothripidae) are predatory on various other insects. These fast moving, ant-mimicking predatory thrips are widely distributed in the tropics, with 17 species described [1,10,11]. In addition, they are unusual among thrips due to the fact that most of them are habitually parthenogenetic and spin a silken cocoon [12]. Moreover, cocoon spinning is observed among the Aeolothripidae [13]. Among *Franklinothrips*, *F. vespiformis* is the most widespread and was noted considerably earlier as distinctive from most of the other Aeolothrips [14]. *F. vespiformis* has gained attention for its potential as a biocontrol agent for a diverse range of greenhouse pests, and it has already been commercially cultured in Europe for certain use [15,16]. *F. orizabensis*, a similar species, has also been documented as a biocontrol agent for thrips management in avocado plantations in California, USA [17].

This review collates the fragmented literature on *Franklinothrips* regarding their global distribution, with a particular focus on host associations and prospects of *F. vespiformis* as a biological control agent. With this review, we anticipate that it may help pave the way for the use of predatory thrips in integrated pest management systems.

## 2. Distribution

### 2.1. F. vespiformis

**Figure 1:**
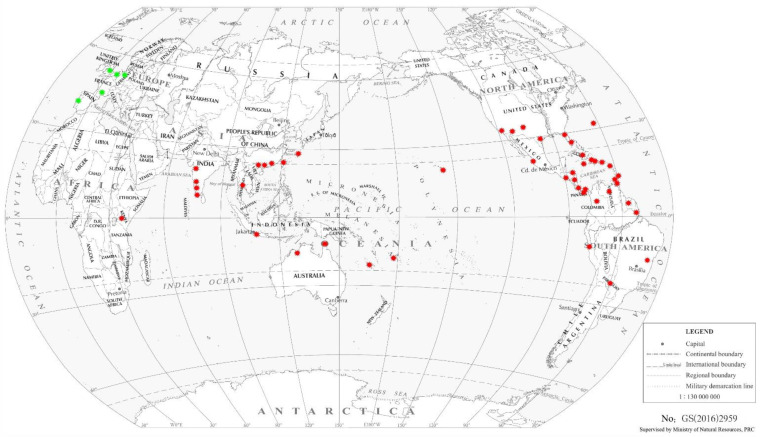
Known global distribution of *Franklinothrips vespiformis*. Red spots indicate locations with wild populations; green spots indicate artificial releases.

The native range is presumed to be Central America [10], although this species has been recorded subsequently in North and South America, Southeast Asia, Africa, Oceania, and Europe. *F. vespiformis* is distributed in different locations with references, as shown in Table 1, and distributed in wild and artificially released populations around the globe, as shown in Figure 1. 

In Latin America, *F. vespiformis* was first described in 1909 [22] and subsequently noted as abundant in avocado agroecosystems in Mexico [23]. This species has been found in Taiwan [32], India [33,34], and many Caribbean, Central, and South American countries, including Nicaragua, Peru, and Brazil [24,25]. Finally, in Oceania, *F. vespiformis* was recorded from Fiji, New Caledonia, as well as the eastern coast of Australia [10], also found in Japan, Thailand and mainland of China [7,11,36]. 

### 2.2. Other Franklinothrips Species

Compared with the pantropical *F. vespiformis*, the reported distribution of 16 other *Franklinothrips* spp. are relatively more localized. The current known distribution includes *F. atlas* Hood and *F. megalops* Trybom (mainly in Africa), *F. basseti* Mound and Marullo as well as *F. variegatus* Girault (Australia), *F. brunneicornis* Mound and Reynaud (New Caledonia), and *F. fulgidus* Hood and *F. lineatus* Hood (Brazil) [1]. Five additional species are noted from Asia, i.e., *F. rarosae* Reyes (Philippines), *F. strasseni* Mound and Reynaud (Nepal), *F. suzukii* Okajima (Taiwan), *F. tani* Mirab-Balou, Shi and Chen (China), and *F. uttarakhandiensis* Vijay Veer (India) [1]. Moreover, three additional species are recorded from Central America, i.e., *F. tenuicornis* Hood (Panama), as well as *F. orizabensis* Johansen and *F. caballeroi* Johansen (Mexico and Costa Rica). Furthermore, in USA, *F. orizabensis*, which closely resembles *F. vespiformis*, has been reported from Arizona, California, Colorado, Florida, and Texas [17,43].

## 3. Morphological Characteristics

**Figure 2:**
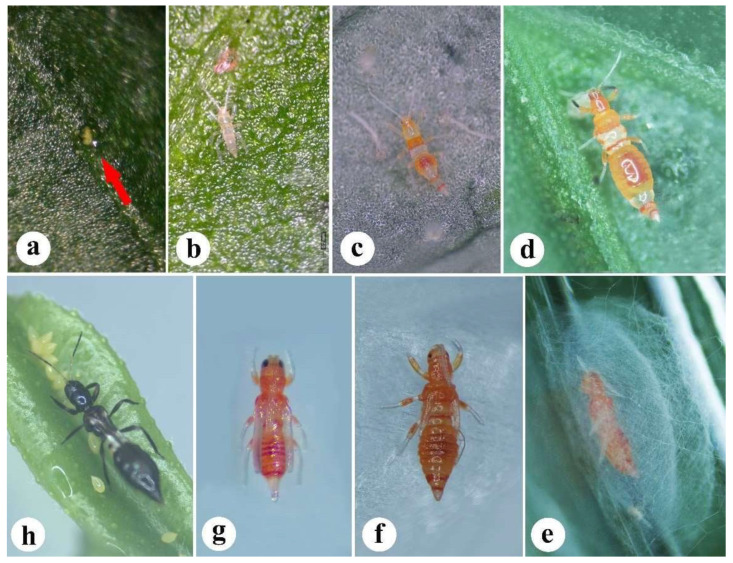
Different stages of *Franklinothrips vespiformis*. (**a**) Egg; (**b**–**d**) Larva: (**b**) Newly emerged larva; (**c**) first instar larva; (**d**) second instar larva; (**e**–**g**) pupal stages: (**e**) pupa into cocoon; (**f**) Pupal stage 1; (**g**) pupal stage 2; (**h**) adult.

*F. vespiformis* experiences partial metamorphosis, developing through egg, larva, pupa, and adult stages (Figure 2). The following is based on the authors’ observations, which is supplemented with published findings [36,44,45].

### 3.1. Eggs

Eggs are produced singly inside the leaf tissue, and they can be distinguished by yellow-green projections. Eggs are kidney-shaped and transparent white, with dimensions of 0.4 ± 0.01 mm by 0.1 ± 0.003 mm (Figure 2a).

### 3.2. Larva

Two instars are included in the larval period. The newly emergent first instars are pale white, with the third antennal segment about 3.5 to 4.5 times as long as wide (Figure 2b). After feeding for 1 or 2 days, the mesothorax and abdomen segments III–VII develop a red coloration (Figure 2c). The second instars have a distinctive hump-back. In addition, the head and prothorax develop a red coloration as the mesothorax. The second instar in the third antennal segment is about 7.0 to 8.0 times as long as wide, and the fore tibia and tarsus are dark (Figure 2d). Both of the instars possess seven segmented antennae with three distal segments, which are closely fused. The red hypodermal pigments are only present on the femora.

### 3.3. Pupa

Pupae are found underneath the leaves, inside a white silk cocoon constructed by the larva (2e). The pupa are red in color with three stages, pre-pupal stage, pupal stage 1 (Figure 2f) and pupal stage 2 (Figure 2g). Wing buds are well developed, but shorter in pre-pupal stage (show non-obvious movement, prepared for cocoon construction). The pupal skin of the appendages is segmented only in pre-pupa. The antennal sheaths do not reach the metathorax (pupa 1), but reach the abdomen (pupa 2). In addition, posterior wing buds reach abdominal segment III (pupa 1), while both the anterior and posterior wing buds reach abdominal segment V (pupa 2). The legs and hind tibiotarsus are shorter than pterothorax (pupa 1), and the legs and hind tibiotarsus are longer than pterothorax (pupa 2).

### 3.4. Adult Female

**Figure 3:**
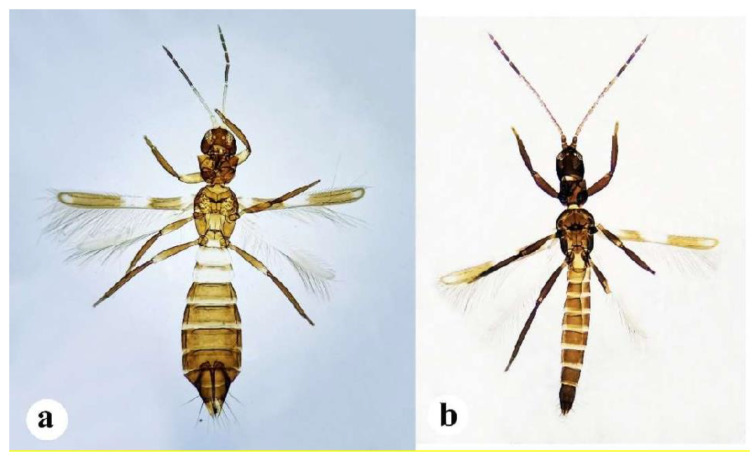
Sexual dimorphism in adult *Franklinothrips vespiformis.* (**a**) Female; (**b**) Male.

Female *F. vespiformis* (myrmici) are common and have a body length of 2.5–3.0 mm (Figure 3a). Females are fully winged and their forewing is slender with a rounded apex. The body is black with white bands on the second and third segments, and an anteriorly narrowed abdomen. The abdomen is broadest at segment five or six. The body, legs, and antennae are brown. However, antennal segments I–III and abdominal segments II and III are yellow. Moreover, the anterior margins are brown and the femora is often yellowish at distal end. Legs brown with femora yellowish at distal end. Fore-wing brown with three paler areas in the base, middle and sub-apex.

### 3.5. Adult Male

Male *F. vespiformis* are rare, similar to female in colour with a smaller and less ant-like appearance (Figure 3b). Males have a longer and darker antennae, a less constricted waist, and commonly paler wings. The second and third antennal segment is approximately as long as the head, with a long sensory metanotum formed of irregular scallops. The head is broader than long, the eyes are prolonged ventrally, and the posterior ocelli are larger than the anterior. The prothorax is narrower towards the base, and the metanotum has no sculpture medially, with long and slender legs. Abdominal sternite II with two pairs of discal setae; sternites III–VIII with two pairs of posteromarginal setae and one pair of discal setae in a line.

## 4. Life History

### 4.1. Developmental Parameters

*F. vespiformis* is active at temperatures over 18 °C and develop from egg to adult within roughly 3 weeks at 27 °C. Moreover, it survives up to 60 days as an adult (Table 2), with no reported diapause. Previous studies of mass storage suggest a differential cold tolerance among the different life stages. In general, the viability of the eggs declines when stored below 7.0 °C, although storing eggs at 12.5 °C for 4–5 weeks was possible [15,16]. The potential to store eggs may assist the mass rearing and dissemination of *F. vespiformis* as a biological control agent.

### 4.2. Sex Ratio

Although *F. vespiformis* consists of both males and females, it is usually an unisexual species. Males were not found in Japan [36] and appear to be rare in populations from other countries [14,20]. Wolbachia-mediated parthenogenesis has been reported in *F. vespiformis* and other thrips species [46,47,48]. Heat and tetracycline treatments appeared to produce male *F. vespiformis*. Despite the fact that males produced motile sperm, which was forwarded via spermatheca, mating had no effect on the subsequent generation’s sex ratios. This indicates that the sperm do not fertilize eggs [46]. Among the introduced thrips, parthenogenesis is common, possibly spreading more easily than sexual forms [49,50].

### 4.3. Ovipositing Behavior

Arakaki and Okajima [36] as well as Arakaki, Miyoshi, and Noda [46] studied the reproductive behavior of *F. vespiformis*. Viable eggs are produced via parthenogenesis with eggs laid singly into the stem, leaf vein or other soft plant tissue using their serrated ovipositor. Females can oviposit three eggs within an hour, producing 150 to 200 eggs in their lifetime. Moreover, females deposit a drop of yellowish protective secretion on the exposed tip of the eggs, which makes them difficult to locate.

### 4.4. Cocoon Spinning

Several species among *Franklinothrips* and *Aeolothrips* construct silken cocoons underneath the leaves or in the soil or leaf litter, for example, *Aeolothrips kuwanaii*, *A. fasciatus*, *A. melaleucus*, *Orothrips kelloggi*, *Ankothrips yuccae*, and *A. gracilis* [10,12,51]. Reyne [12] indicated that the cocoon production of *F. vespiformis* takes a full and larvae were observed sharply twisting and turning their abdomens, with the final cocoon as white and oval-shaped, measuring roughly 2.7 mm × 1.3 mm in size [12,36].

### 4.5. Ant-Mimicking Behavior

While some degree of myrmecomorphy is associated with most of the *Franklinothrips*, the extent of ant-like features and behavior is highly pronounced in adult female *F. vespiformis* [14]. A highly constricted first abdominal segment produces an ant-like waist [36]. Similar to ants, individuals can run quickly and palpate their antennae on the ground. These distinguishing characteristics have been proposed in order to help adults escape predation [52,53].

## 5. *Franklinothrips*: Perspectives for Biological Control of Pests

### 5.1. Natural Prey Range

*Franklinothrips* are generalist or opportunist feeders of thrips, but also attack a broad range of small arthropods and smaller conspecifics [10]. Among them, *F. vespiformis* is known to prey upon phytophagous insects and mites from several orders (Table 3). Larvae and adults move quickly and seize the prey with their front legs, which are used to hold the prey while feeding [36].

Both larvae and adults are particularly predacious on other thrips, feeding on adults, larvae, and pupal stages. In Trinidad, *F. vespiformis* was observed feeding on the cacao thrips, *Selenothrips rubrocinctus* and *Dinurothryps hookeri* on ornamental flowers, and *Caliothrips insularis* on the Sudan grass [26]. In Mexico, *F. vespiformis* are the most prevalent among 16 predatory thrips, which were captured in an avocado orchard infested with several species of pest thrips. In Brazil, *F. vespiformis* was frequently found in association with *Leucothrips furcatus*, which appeared as the prey [31]. Additional economically important thripine targets for *F. vespiformis* include *Thrips tabaci*, *T. palmi*, and *Frankliniella occidentalis* [40].

In addition, *F. vespiformis* is predaceous on several non-thripine targets. In citrus and avocado plantation in Central and South America, *F. vespiformis* naturally prey on spider mites (*Oligonychus yothersi*), leafhoppers (*Idona minuenda*), and whiteflies (*Trialeurodes floridensis*) [29]. In laboratory tests, *F. vespiformis* was observed piercing serpentine leafminer (*Liriomyza trifolii*) larvae. However, nymps of the cotton aphid, *Aphis gossypii* were found to be unsuitable as prey [36].

In nature, *F. vespiformis* is usually found on low growing plants, shrubs, and bushes and has similar feeding habits to *Aeolothrips melaleucus* [51,54]. It is found in a variety of habitats, including roadsides [54], rainforests, orchards, and field crops [40]. Adult *Franklinthrips* feed on non-prey materials and can survive for extended periods of time on pollen and plant sap [43]. When reared under crowded conditions (10 individuals/Petri dish), adults and larval become cannibalistic [36].

**Table 3:** Known prey and associated host plant associations for *Franklinothrips vespiformis*.

Prey	Species	Stage of Prey *	Host Plant	Reference(s)
Leafhopper	*Idona minuenda*	na	Citrus, avocado	[29]
Leafminer	*Liriomyza trifolii*	L	Chrysanthemums and celery	[36]
Spider mites	*Oligonychus yothersi*	E, L, A	Solanaceous plants	[29]
	*Tetranychus* *urticae*	L, A	Laboratory	[36,41]
	*Tetranychus* *neocaledonicus*	L, A	Lima beans	[30]
Thrips	*Caliothrips* *insularis*	-	Mint	[26]
	*Caliothrips phaseoli*	L, A	Lima bean	[30]
	*Dinurothrips hookeri*	-	Ornamental flowers	[26]
	*Echinothrips americanus*	L, A, P	Greenhouse crops	[40]
	*Frankliniella* *occidentalis*	L, A, P	Apple, vegetables, and ornamental crops	[40,41,55]
	*Frankliniella* *intonsa*	-	Vegetables and ornamental crops	[55,56]
	*Heliothrips haemorrhoidalis*	-	Avocado	[40,57]
	*Leucothrips* *furcatus*	L, A	Curcubits	[31]
	*Parthenothrips dracaenae*	L, A, P	Ficus species, dracaena, [40] palm, and orchid	[40]
	*Scirtothrips* *dorsalis*	L	Chilli and beans	[53]
	*Selenothrips* *rubrocinctus*	-	Sudan grass	[26]
	*Thrips palmi*	L, A	Laboratory	[36,40]
	*Thrips tabaci*	-	Onion	[38,39,40,56]
Whitefly	*Trialeurodes floridensis*	E, L	Citrus, avocado	[29]
	*Bemisia tabaci* (MEAM 1)	-	Laboratory	[36,53]

* E: Egg; L: Larvae; P: Pre-pupa/pupa; A: Adult.

### 5.2. Franklinothrips: As Augmentative Biological Control Agents

While predatory thrips are considered an important component of natural and agro-ecosystems, their use as commercial bio-control agents has gained relatively little attention. However, in Europe, *F. vespiformis* has been tested and marketed for use against thrips in greenhouses, nurseries, botanical gardens, and interiorscapes for many years [15,16,42]. In addition, *F. orizabensis* has been the focus of successful research with an augmentative release against several pest thrips in California avocado groves [17,43].

### 5.3. Release of F. vespiformis in Greenhouse Crops

Several studies have investigated the use of *F. vespiformis* to manage thrips in green- houses. In southern France, *F. vespiformis* was first introduced in rose greenhouses for 2 years, with a predatory mite *Neoseiulus cucumeris* (Acari: Phytoseiidae) to suppress onion thrips *T. tabaci* and western flower thrips *F. occidentalis* [39]. Although widely used, *N. cucumeris* is less effective where temperatures are high. Therefore, *F. vespiformis* was used to supplement the control. In this case, *F. vespiformis* was generally effective at reducing populations of thrips below economic thresholds, although it did not provide a long-term establishment, suggesting that repeated introductions would be needed. Follow-up tests showed that the combined use of *F. vespiformis* and *N. cucumeris* during periods of high thrips infestation gave better results when compared with *N. cucumeris* alone [38]. In another study in greenhouse cucumbers, the weekly release of one *F. vespiformis* adult per plant over 4 weeks after flowering, reduced the populations of thrips on leaves to low levels, although less control was observed on flowers [56]. In Japan, *F. vespiformis* was released in greenhouses to control *T. palmi* in eggplants and cucumbers [36,46].

Some evidence suggests that the releases were successful in ornamental plants. When *F. vespiformis* was used in Crown-of-Thorns (*Euphorbia milii* var. splendens), *Frankliniella occidentalis* larvae were drastically decreased compared to the control. Only one *F. occidentalis* larva/flower was found 7 weeks following the release of *F. vespiformis*, compared to 14 larvae/flower in the control plots [48]. Despite these limited studies, the prey range, choice, and requirements for the release of *F. vespiformis* in most greenhouse crops are yet unknown.

### 5.4. Commercial Availability

Since predatory thrips typically do not occur at high densities, they can be reared for augmentative use. In Europe, *F. vespiformis* is sold through at least two distributors for pest thrips, including *Frankliniella occidentalis*, *Echinothrips americanus*, *Parthenothrips dracaenae*, *Scirtothrips* spp, and *Thrips palmi* [58,59]. The product is sold as adult thrips in a tube, which can be stored for 2 days at 10–15 °C. The success of releases will vary, based on the release rate, environmental conditions, and economic threshold of the target pest [16,17].

## 6. Other Predatory Thrips and Prey Associations

Surveys have found predatory thrips on many natural and cultivated plants, which reflect the host range of their prey (Table 4). Surveys of annual and perennial field crops, shrubs or trees, and roadside vegetation and weeds in three districts of northern Thailand revealed 10 species of predatory thrips in five genera of the Phlaeothripidae, i.e., *Aleurodothrips* *fasciapennis*, *Androthrips flavipes*, *A. ramachandrai*, *Karnyothrips flavipes*, two indeterminate *Karnyothrips* spp., *Leptothrips* sp., *Podothrips lucasseni*, and two indeterminate *Podothrips* spp. In this case, thrips hosts were present throughout the year. Preys of predatory thrips were identified on asteraceous weeds, including *Bidens pilosa* and *Tridax procumbens*, but also eriophyid mites were identified on Siam weed (*Chromolaena odoratum*). *P. lucasseni* was found on eriophyid mites on *Sandoricum koetjape* (santol) and *Litchi chinensis* (litchi). In addition, distinct distributions were found, i.e., *Karnyothrips flavipes* was usually correlated with green field crops, such as coffee, garlic (*Allium sativum*), and Spanish needle (*B. pilosa*). *Kar nyothrips* were observed feeding on unidentified crambid larvae (Lepidoptera: Crambidae) on *Spondias pinnata* (Lepidoptera: Crambidae) in another province. *Podothrips* spp. were observed in association with thrips on *Argyreia capitiformis*, aphids on *Lepistemon bi nectariferum* and *Bidens pilosa*, spider mites on *Bambusa* sp. (bamboo). Childers and Nakahara (2006) [60] found diverse predatory species of thrips in citrus groves in Florida, USA, associated with six weed species. The potential host diversity of predatory thrips on commercial crops highlighted by these surveys suggests that other species could be explored further as biological control agents.

## 7. Future Research Perspectives

Since its original description by Crawford [22], reports show that *F. vespiformis* is a widespread and important natural enemy of thrips and other small arthropods, as well as an interesting and unusual model organism for Batesian mimicry. From a pest management perspective, the advantages of *F. vespiformis* include the relatively wide range of attacked hosts and life-stages. The ability of this predator to attack thrips species, such as *E. americanus*, which are not easily controlled by most of the current commercial biological control agents, including predatory mites [66], is of particular benefit. However, the relatively slow intrinsic rate of increase, when compared with predatory mites [67], and its tendency for cannibalism [36] are hindrances, increasing the cost and complexity for mass production. Nevertheless, as the global market for predatory insects expands and moves towards the use of multiple biological control agents and bioactive molecules [68], we anticipate increased interest, production, and deployment of *F. vespiformis* (and likely *F. orizabensis*) in crops where the current biocontrol agents do not provide reliable (or need supplemental) control. Their ability to feed on eggs of thrips, which are hidden in plant tissues and cryptic prey, such as leafminers [36,69] is also encouraging. Moreover, given that *F. vespiformis* can be cold-stored for a relatively long time period [15] will benefit its distribution to end users.

Despite their potential, it remains unclear on which crops *F. vespiformis* can be most effectively employed. The research gaps identified while compiling this review include the determination of optimal pest/crops associations, and the potential intraguild interactions with other biocontrol agents. To that end, most of the effective application rates and timing need further assessment under both greenhouse and field conditions. The provision of thrips banker plant systems or other supplemental food, such as pollen also need further investigation [40]. Of note, *F. vespiformis* may feed on a commercial supply of decapsulated brine shrimp eggs, which are used to support other commercially produced beneficial insects and mites (SPA personal observations). Moreover, it is possible that the ant-like appearance of *F.* *vespiformis* may help in the protection from negative intraguild interactions, while its adaption to topical environments may make it less likely to be established outdoor in temperate regions.

In conclusion, *F. vespiformis* is both a charismatic and economically important species of thrips, which warrants further attention in both conservation and augmentative biological control research. Furthermore, additional ecological and applied pest management studies will determine the role of this predator as both an invasion risk in natural ecosystems and as a commercial success in agricultural pest control.

## Figures and Tables

**Table 1:** The distribution of *Franklinothrips vespiformis*.

Region	Country (Location)	Reference(s)
North America	USA (Colorado)	[18]
	USA (Arizona, California, Florida, Texas)	[10,19,20,21]
	Mexico	[22,23,24,25]
Caribbean	Jamaica, Dominican Republic, Barbados	[24,25]
	Puerto Rico	[25]
	Trinidad and Tobago	[26,27]
	St. Vincent Island, West Indies	[24,25]
Central America	Costa Rica, El Salvador, Nicaragua	[14,22,24]
	Honduras	[28]
	Panama	[14]
South America	Brazil	[29,30,31]
	Paraguay	[24]
	Peru (Miraflores)	[14]
	Surinam	[12,25]
Asia	China (Taiwan)	[32]
	China (Guangdong Yunnan, Guangxi)	[11]
	India (Karnataka, Maharashtra, Kerala, Tamil Nadu)	[33,34]
	Indonesia (Java)	[35]
	Japan (Okinawa)	[36,37]
	Thailand	[7,25]
**Oceania**	Australia (Queensland)	[10]
	New Caledonia	[10]
	Hawaii	[24]
Europe	France	[38,39,40]
	Germany	[41]
	Portugal	[24]
	UK	[15,16,42]

**Table 2:** Developmental parameters (days) of *Franklinothrips vespiformis* reared at different temperatures.

Development Parameter	Temperature (°C)			Reference(s)
	21 °C	25 °C	27 °C	
Life Stage (Days (±SE))				[15,16]
Eggs	16.06 ± 0.8	10.39 ± 0.1	9.7 ± 0.0	
Larva 1	4.04 ± 0.12	2.03 ± 0.0	1.9 ± 0.0	
Larva 2	3.9 ± 0.1	2.1 ± 0.0	1.1 ± 0.0	
Prepupal and Pupal	12.5 ± 0.1	7.4 ± 0.1	5.3 ± 0.0	
Unmated Males	24.3 ± 1.6	16.4 ± 1.3	9.0 ± 0.7	
Mated Males	15.6 ± 1.9	12.8 ± 1.6	8.0 ± 0.6	
Reproductive parameter (±SE)				
Unmated Females				
Pre-oviposition period (days)	1.6 ± 0.2	0.9 ± 0.2	2.4 ± 1.0	
Mean total progeny	67.9 ± 21.4	71.2 ± 12.9	8.5 ± 3.8	
Mean daily progeny	2.3 ± 0.2	4.1 ± 0.3	1.0 ± 0.1	
Mean lifetime oviposition rate	154 ± 22.4	314 ± 44.1	105 ± 17.9	
Mean daily oviposition rate	7.1 ± 0.4	18.1 ± 13.6	12.9 ± 1.3	
Mated Females				
Pre-oviposition period (days)	1.5 ± 0.2	0.9 ± 0.1	0.8 ± 0.2	
Mean total progeny	35.2 ± 6.6	44.4 ± 11.8	8.4 ± 2.8	
Mean daily progeny	1.8 ± 0.1	3.1 ± 0.2	0.9 ± 0.2	
Mean lifetime oviposition rate	128 ± 25.5	220 ± 47.9	101 ± 14.7	
Mean daily oviposition rate	6.5 ± 0.5	15.9 ± 1.1	12.8 ± 1.1	
Population growth parameters (±SE)				
Net reproductive rate (R_0_)	18.5 ± 0.18	33.3 ± 0.28	4.5 ± 0.07	
Generation time (T_c_)	49.1 ± 0.12	27.9 ± 0.05	24.2 ± 0.06	
Intrinsic rate of increase (r_m_)	0.06 ± 0.0002	0.13 ± 0.0003	0.06 ± 0.0007	
Finite rate of increase (λ)	1.06 ± 0.0002	1.14 ± 0.0004	1.07 ± 0.0007	
Survival time in days (T_d_)	11.12 ± 0.03	5.16 ± 0.01	11.05 ± 0.12	

**Table 4:** Host plant associations of *Franklinothrips* and other predatory thrips with their prey.

Predatory Thrips	Prey	Host Plant	Country	Reference(s)
*Aleurodothrips* *fasciapennis*	*Thrips* spp., Coccus sp., *Aleurodicus dispersus*	*Bidens pilosa* (Asteraceae), *Derris indica* (Fabaceae), *Spondias pinnata* (Anacardiaceae), *Manihot esculenta* (Euphorbiaceae)	Thailand	[7]
	Whiteflies, psyllid, scale	Citrus (Rutaceae)	Florida, USA, China	[60,61]
	*Planococcus citri*	Grape (Vitaceae)	Iran	[62]
*Androthrips* *flavipes*	*Gynaikothrips ficorum*	*Ficus retusa* (Moraceae)	Thailand	[7]
	*Liothrips karnyi*	*Piper nigrum* (Piperaceae)	Kerala, India	[63]
*A. ramachandrai*	*Montandoniola confusa*	*Pterocarpus indicus* (Leguminosae)	Thailand	[7]
	*Gynaikothrips uzeli*	*Ficus retusa* (Moraceae)	China	[62]
	*G. uzeli*	*Ficus benjamina* (Moraceae)	Argentina	[64]
*F. vespiformis*		Citrus spp., Avocado	Central and South America	[29]
*F. orizabensis*		Avocado, *Persea americana* (Lauraceae)	California, USA	[43]
*Karnyothrips* *flavipes*	*Coccus viridis*	*Coffea arabica* (Rubiaceae)	Thailand	[7]
	*Hypothenemus hampei*	*Coffea arabica* (Rubiaceae)	Kenya	[65]
	*Thrips* spp.	Citrus (Rutaceae)	Florida, USA	[61]
	*Thrips* spp.	*Allium sativum* (Alliaceae), *Bidens pilosa* (Asteraceae), *Gomphrena celosiodes* (Amaranthaceae)	Thailand	[7]
*Leptothrips* spp.	*Thrips* spp.	*Bidens pilosa*, *Tridax procumbens*, *Chromolaena* *odoratum* (Asteraceae)	Thailand	[7]
	*Thrips* spp.	Citrus (Rutaceae)	Florida, USA	[61]
*Podothrips* spp.	*Aceria litchi*, *Thrips* spp., *Aphis* sp., *Eutetranychus* sp.	*Sandoricum koetjape* (Meliaceae), *Litchi chinensis* (Sapindaceae) *Argyreia capitiformis* (Convolvulaceae), *Bambusa* sp. (Poaceae)	Thailand	[7]

## Data Availability

Not applicable.

## References

[1] Roskov Y., Ower G., Orrell T., Nicolson D., Bailly N., Kirk P.M., Bourgoin T., DeWalt R.E., Decock W., Nieukerken E., ThripsWiki (2019). Providing information on the World’s thrips (version Nov 2018). Species 2000 & ITIS Catalogue of Life, 2019 Annual Checklist.

[2] Parker B., Skinner M., Ewis T. (1995). Thrips Biology and Management.

[3] Riley D.G., Joseph S.V., Srinivasan R., Diffie S. (2011). Thrips vectors of tospoviruses. J. Integr. Pest Manag..

[4] Morse J.G., Hoddle M.S. (2006). Invasion biology of thrips. Annu. Rev. Entomol..

[5] Reynaud P. (2010). *Thrips* (Thysanoptera). Chapter 13.1. BioRisk.

[6] zur Strassen R., Parker B., Skinner M., Ewis T. (1995). Binomial data of some predacious thrips. Thrips Biology and Management.

[7] Saengyot S. (2016). Predatory thrips species composition, their prey and host plant association in Northern Thailand. Agric. Nat. Resour..

[8] Parrella M., Rowe D., Horsburgh R. (1982). Biology of *Leptothrips mali*, a common predator in Virginia apple orchards. Ann. Entomol. Soc. Am..

[9] Haviland D.R., Rill S.M., Gordon C.A. (2021). Field biology of *Scolothrips sexmaculatus* (Thysanoptera: Thripidae) as a predator of *Tetranychus pacificus* (Acari: Tetranychidae) in California almonds. J. Econ. Entomol..

[10] Mound L.A., Reynaud P. (2005). *Franklinothrips*; a pantropical Thysanoptera genus of ant-mimicking obligate predators (Aeolothripidae). Zootaxa.

[11] Mirab-Balou M., Shi M., Chen X.-X. (2011). A new species of *Franklinothrips Back* (Thysanoptera: Aeolothripidae) from Yunnan, China. Zootaxa.

[12] Reyne A. (1920). A Cocoonspinning Thrips. Tijdschr. Voor Entomol..

[13] Pereyra V., Cavalleri A. (2012). The genus *Heterothrips* (Thysanoptera) in Brazil, with an identification key and seven new species. Zootaxa.

[14] Goldarazena A., Gattesco F., Atencio R., Korytowski C. (2012). An updated checklist of the Thysanoptera of Panama with comments on host associations. Check List.

[15] Larentzaki E., Powell G., Copland M.J. (2007). Effect of cold storage on survival, reproduction and development of adults and eggs of *Franklinothrips vespiformis* (Crawford). Biol. Control.

[16] Larentzaki E., Powell G., Copland M.J. (2007). Effect of temperature on development, overwintering and establishment potential of *Franklinothrips vespiformis* in the UK. Entomol. Exp. Appl..

[17] Hoddle M.S., Oevering P., Phillips P.A., Faber B.A. (2004). Evaluation of augmentative releases of *Franklinothrips orizabensis* for control of *Scirtothrips perseae* in California avocado orchards. Biol. Control.

[18] Mahaffey L.A., Cranshaw W.S. (2010). Thrips species associated with onion in Colorado. Southwest. Entomol..

[19] Stannard L.J. (1952). Peanut-winged thrips (Thysanoptera: Thripidae). Ann. Entomol. Soc. Am..

[20] Stannard L.J. (1952). Phylogenetic studies of *Franklinothrips* (Thysanoptera: Aeolothripidae). J. Wash. Acad. Sci..

[21] Hoddle M.S., Mound L.A., Paris D.L. (2012). Thrips of California.

[22] Crawford D. (1909). Some Thysanoptera of Mexico and the south. I. Pomona Coll. J. Entomol..

[23] Cambero-Campos J., Johansen-Naime R., García-Martínez O., Cerna-Chávez E., Robles-Bermúdez A., Retana-Salazar A. (2011). Species of thrips (Thysanoptera) in avocado orchards in Nayarit, Mexico. Fla. Entomol..

[24] CABI (2021). Invasive Species Datasheet, *Franklinothrips vespiformis* (Vespiform Thrips). https://www.cabi.org/isc/datasheet/24485.

[25] Greathead D.J., Greathead A.H. (1992). Biological control of insect pests by insect parasitoids and predators: The BIOCAT database. Biocontrol.

[26] Callan E.M. (1943). Natural enemies of the cacao thrips. Bull. Entomol. Res..

[27] Williams C. (1918). Plant diseases and pests: Notes on some Trinidad thrips of economic importance. Trinidad Tobago Bull..

[28] Watson J., Hubbell T. (1924). On a collection of Thysanoptera from Honduras. Fla. Entomol..

[29] Moulton D. (1932). The Thysanoptera of South America. Revta Entomol..

[30] de França S.M., de Melo Júnior L.C., Neto A.V.G., Silva P.R.R., Lima É.F.B., Melo J.W.S. (2018). Natural enemies associated with *Phaseolus lunatus* L. (Fabaceae) in Northeast Brazil. Fla. Entomol..

[31] Lima E.F.B., Souza-Filho M.F. (2018). *Leucothrips furcatus* (Thysanoptera Thripidae): A new pest of *Sechium edule* (Cucurbitaceae) in Brazil. Bull. Insectol..

[32] Wang C.-L. (1993). Two new records and two new species of thrips (Thysanoptera, Terebrantia) of Taiwan. Chin. J. Entomol..

[33] Tyagi K., Kumar V. (2016). Thrips (Insecta: Thysanoptera) of India-an updated checklist. Halteres.

[34] Mahendran P., Radhakrishnan B. (2019). *Franklinothrips vespiformis* Crawford (Thysanoptera: Aeolothripidae), a potential predator of the tea thrips, *Scirtothrips bispinosus* Bagnall in south Indian tea plantations. Entomon.

[35] Sartiami D., Mound L.A. (2013). Identification of the *terebrantian thrips* (Insecta, Thysanoptera) associated with cultivated plants in Java, Indonesia. ZooKeys.

[36] Arakaki N., Okajima S. (1998). Notes on the biology and morphology of a predatory thrips, *Franklinothrips vespiformis* (Crawford) (Thysanoptera: Aeolothripidae): First record from Japan. Entomol. Sci..

[37] Pijnakker J., Overgaag D., Guilbaud M., Vangansbeke D., Duarte M., Wäckers F. (2019). Biological control of the Japanese flower thrips *Thrips setosus* Moulton (Thysanoptera: Thripidae) in greenhouse ornamentals. IOBC-WPRS Bull..

[38] Pizzol J., Nammour D., Hervouet P., Poncet C., Desneux N., Maignet P. Population dynamics of thrips and development of an integrated pest management program using the predator *Franklinothrips vespiformis*. Proceedings of the XXVIII International Horticultural Congress on Science and Horticulture for People (IHC2010).

[39] Pizzol J., Nammour D., Ziegler J.-P., Voisin S., Maignet P., Olivier N., Paris B. Efficiency of *Neoseiulus cucumeris* and *Franklinothrips vespiformis* for controlling thrips in rose greenhouses. Proceedings of the International Symposium on High Technology for Greenhouse System Management: Greensys 2007.

[40] Loomans A., Vierbergen G. (1999). *Franklinothrips*: Perspectives for greenhouse pest control. IOBC WPRS Bull..

[41] Zegula T., Sengonca C., Blaeser P. (2003). Entwicklung, reproduktion und Prädationsleistung von zwei Raubthrips-arten *Aeolothrips intermedius* Bagnall und *Franklinothrips vespiformis* Crawford (Thysanoptera: Aeolothripidae) mit ernährung zweier natürlicher beutearten. Gesunde Pflanz..

[42] Cox P., Matthews L., Jacobson R., Cannon R., MacLeod A., Walters K. (2006). Potential for the use of biological agents for the control of *Thrips palmi* (Thysanoptera: Thripidae) outbreaks. Biocontrol Sci. Technol..

[43] Hoddle M.S. (2003). Predation behaviors of *Franklinothrips orizabensis* (Thysanoptera: Aeolothripidae) towards *Scirtothrips perseae* and *Heliothrips haemorrhoidalis* (Thysanoptera: Thripidae). Biol. Control.

[44] Tyagi K., Mound L., Kumar V. (2008). Sexual dimorphism among Thysanoptera Terebrantia, with a new species from Malaysia and remarkable species from India in Aeolothripidae and Thripidae. Insect Syst. Evol..

[45] Kumar B., Omkar (2021). Thrips. Polyphagous Pests of Crops.

[46] Arakaki N., Miyoshi T., Noda H. (2001). Wolbachia-mediated parthenogenesis in the predatory thrips *Franklinothrips vespiformis* (Thysanoptera: Insecta). Proc. R. Soc. London Ser. B Biol. Sci..

[47] Kumm S., Moritz G. (2008). First detection of Wolbachia in arrhenotokous populations of thrips species (Thysanoptera: Thripidae and Phlaeothripidae) and its role in reproduction. Environ. Entomol..

[48] Nguyen D.T., Spooner-Hart R.N., Riegler M. (2016). Loss of Wolbachia but not Cardinium in the invasive range of the Australian thrips species, Pezothrips kellyanus. Biol. Invasions.

[49] O’Neill K. (1960). Identification of the Newly Introduced *Phlaeothripid Haplothrips*? Clarisetis Priesner (Thysanoptera). Ann. Ent Soc. Am..

[50] Pal S., Wahengbam J., Raut A., Banu A.N. (2019). Eco-biology and management of onion thrips (Thysanoptera: Thripidae). J. Entomol. Res..

[51] Putman W.L. (1942). Notes on the predaceous thrips *Haplothrips subtilissimus* hal. and Aeolothrips melaleucus Hal. Can. Entomol..

[52] Johansen R.M. (1977). Algunos aspectos sobre la conducta mimetic de *Franklinothrips vespiformis* (Crawford (Insecta: Thysanoptera)). An. Instit. Biol. Univ. Nac. Mex..

[53] Johansen R.M. (1983). Nuevos estudios acerca del mimetismo en el genero *Franklinothrips* Back (Insect: Thysanoptera) en Mexico. An. Inst. Biol. Univ. Nac. Mex..

[54] Mao R., Xiao Y., Arthurs S. (2018). Vespiform Thrips Franklinothrips vespiformis Crawford (Insecta: Thysanoptera: Aeolothripidae).

[55] Kort I.B., Moraza M.L., Attia S., Mansour R., Kheder S.B. (2020). Beneficial arthropods as potential biocontrol candidates of thrips (Thysanoptera: Thripidae) occurring in Tunisian citrus orchards. Biologia.

[56] Imura T. (2003). Potential for biological control of thrips on greenhouse cucumbers by *Franklinothrips vespiformis* (Crawford). Proc. Kansai Plant Prot. Soc..

[57] McMurtry J.A. (1992). The role of exotic natural enemies in the biological control of insect and mite pests of avocado in California. Proceedings of the Second World Avocado Congress, Orange, CA, USA, 21–26 April 1991.

[58] Entocare (2021). Predatory Thrips *Franklinothrips vespiformis*. https://entocare.nl/biological-control-thrips/franklinothrips-vespiformis/?lang=en.

[59] Koppert (2021). *Franklinothrips vespiformis*, Thrips Prédateur *Franklinothrips vespiformi*. https://www.koppert.fr/franklinothrips-vespiformis/.

[60] Childers C.C., Nakahara S. (2006). Thysanoptera (thrips) within citrus orchards in Florida: Species distribution, relative and seasonal abundance within trees, and species on vines and ground cover plants. J. Insect Sci..

[61] Hua L.Z. (2000). List of Chinese Insects: Homoptera: Adelgoidea and Aphidoidea.

[62] Mirab-balou M., Chen X.X. (2012). *Aleurodothrips fasciapennis* Franklin: A newly recorded genus and species for Iran (Thysanoptera: Phlaeothripidae). Munis Entomol. Zool..

[63] Devasahayam S., Koya K.M.A. (1994). Natural enemies of major insect pests of black pepper (*Piper nigrum* L.) in India. J. Spices Arom. Crop..

[64] de Borbon C.M., Agostini J.P. (2011). *Gynaikothrips uzeli* (Zimmermann) and *Androthrips ramachandrai* Karny (Thysanoptera: Phlaeothripidae), first record for Argentina. Rev. Faculdad Cienc. Agrar. Univ. Nac. Cuyo..

[65] Vega F.E., Infante F., Castillo A., Jaramillo J. (2009). The coffee berry borer, *Hypothenemus hampei* (Ferrari) (Coleoptera: Curculionidae): A short review, with recent findings and future research directions. Terr. Arthropod Rev..

[66] Ghasemzadeh S., Leman A., Messelink G.J. (2017). Biological control of *Echinothrips americanus* by phytoseiid predatory mites and the effect of pollen as supplemental food. Exp. Appl. Acarol..

[67] Nomikou M., Janssen A., Schraag R., Sabelis M.W. (2001). Phytoseiid predators as potential biological control agents for *Bemisia tabaci*. Exp. Appl. Acarol..

[68] Hussain M., Debnath B., Qasim M., Bamisile B.S., Islam W., Hameed M.S., Wang L., Qiu D. (2019). Role of saponins in plant defense against specialist herbivores. Molecules.

[69] Sureshkumar N., Ananthakrishnan T.N. (1987). Biotic interactions in relation to prey-predator relationship with special reference to some thrips species (Thysanoptera: Insecta). J. Entomol. Res..

